# Parental deprivation due to death in male soldiers with psychiatric disorders

**DOI:** 10.4103/0019-5545.43049

**Published:** 2005

**Authors:** M.S.V Kama Raju, Kalpana Srivastava, M. Diwakar, P.S. Bhat

**Affiliations:** * Commandant and Professor of Psychiatry, Military Hospital, Kirkee, Pune; ** Scientist ‘D’, Department of Psychiatry, Armed Forces Medical College, Pune; *** Graded Specialist (Psychiatry), Command Hospital, Lucknow; **** Classified Specialist (Psychiatry), 92 BH C/O 56 APO

**Keywords:** Parental loss, psychiatric disorders, bipolar disorder, alcohol dependence syndrome

## Abstract

**Background:**

The importance of early relationship with parents has been stressed by most personality theorists. Deprivation of the nurturing influence can lead to an adverse impact.

**Aim:**

To study the effect of early parental loss in the development of adult psychiatric disorder.

**Methods:**

A total of 289 soldiers suffering from assorted ICD-10 identified psychiatric disorders were studied to ascertain parental losses during their developmental period. The findings were compared with those of 127 patients drawn from general medical wards.

**Results:**

A higher percentage of psychiatric patients had lost their parents before the age of 18 years compared with medical patients (21.5% vs. 8.7%). The difference, which was highly significant, was due to bipolar disorder to some extent and alcohol dependence syndrome to a larger extent. Loss of the father appears to be more significant than loss of the mother. Parental loss is found to be not a significant factor in depression and neurotic disorders. There is no excess of maternal loss in cases of depression.

**Conclusion:**

This study indicates that parental loss is a significant factor in the future development of psychiatric disorders. It does not appear to be an important factor in the development of neurotic disorders. The aspect requires comprehensive evaluation.

## INTRODUCTION

Most personality theorists emphasize the importance of early relationships with parents. Deprivation of this crucial nurturing influence due to death, desertion or divorce can be expected to have an adverse impact on the developing organism. Many workers have attempted to ascertain the risk that early parental loss imposes on nascent personalities for the development of psychopathology in adulthood. Wahl *et al*.^1^ reported significantly more parental deaths in childhood of 568 men of the US Navy who suffered from schizophrenia. Brown *et al*.^2^ found that loss of the mother before 11 years of age was associated with a greater risk for depression in adulthood. In a study of 1018 female twins, Kendler *et al*.^3^ found that parental death in childhood was significantly associated with panic disorder in adulthood. Dennehy^4^ studied parental death in 1020 psychiatric patients and compared the results with the national census data of the UK. A significant excess of paternal deaths was noted in depressives of both the sexes, male schizophrenia patients, male alcoholics and male drug addicts. Significant excess of mother loss was also found in male schizophrenia patients, male depressive patients and alcoholic patients of both the sexes. Hilgrad *et al*.^5^ compared early parental death in 929 male alcoholic patients with that of 1096 persons from the community and found that alcohol-dependent patients had significantly more paternal and maternal deaths in childhood. However, discrepant findings have also been reported. Granville-Grossman^6^ in a study of 1252 patients with schizophrenia found that bereavement *per se* did not increase the morbid risk for schizophrenia. Pitts *et al*.^7^ compared parental deaths in 748 psychiatric patients with matched controls from general wards. They concluded that deprivation had no independent role in the genesis of psychiatric illness. Tennent *et al*.^8^ found that neither maternal nor paternal death before 18 years of age was associated with subsequent psychiatric disorders. In a study of 85 patients with the alcohol dependence syndrome, Furukawa *et al*.^9^ found no significant increase in paternal or maternal deaths. Wig *et al*.^10^ found that 33% of parental losses during the development period of psychiatric patients was accounted for by affective disorders. In the only study so far in the armed forces setting, Das^11^ investigated parental losses in 203 psychiatric patients consisting of 109 patients with neuroses (53.7%), 52 with schizophrenia (26.67%), 23 with affective disorders (11.33%) and 1 with alcoholism (0.5%); 34% of the psychiatric patients and 27.6% of the medical patients had suffered parental losses before 16 years of age. The difference, however, was not statistically significant; 25% of the parental losses in his sample were accounted for by patients with affective disorders. Over the years, psychiatric diagnoses have become more specific due to operationalized diagnostic criteria. There are fewer cases of neuroses now and the prevalence of alcoholism has increased. In view of these developments, a fresh evaluation appeared overdue.

## METHODS

Two hundred eighty-nine patients admitted to various psychiatric centres were selected at random for the study. All patients were persons below officer rank (PBOR) and male. The sample consisted of 66 patients with schizophrenia (22.8%), 22 with bipolar disorder (76%), 46 with depression (15.9%), 25 with unspecified psychosis (8.65%), 40 with neurotic disorders (13.8%), 56 with alcohol dependence syndrome (19.4%) and 34 patients with miscellaneous conditions (11.8%). Another 127 medical and surgical patients who had no current or life-time psychiatric illness were selected at random as controls. All the patients were assessed by conventional clinical psychiatric evaluation and diagnoses were made as per the ICD-10 criteria. As defined by Tennent and Bernardi,^12^ age up to 18 years was considered as the developmental period. There was no significant difference between the study sample and controls in respect of age (mean age: 32.78 vs. 34.1 years; Z=0.85 [NS]), education in years (mean: 10.35 vs. 10.45 years; Z=0.27 [NS]) and marital status (married: 65 vs. 73; χ^2^ =1.69 [NS]).

## RESULTS

In general, psychiatric patients suffered a significantly high number of parental deaths in their formative years when compared to medical patients (21.45% vs. 8.66%). Both maternal (7.26% vs. 1.57%) and paternal deaths (14.19% vs. 7.08%) were significantly in excess in psychiatric patients (Table 1).

**Table 1:** Parental death in medical and psychiatric patients before the age of 18 years

	Loss of both the parents *n* (%)	Loss of father *n* (%)	Loss of mother *n* (%)
General medical condition (*n*=127)	11 (8.66)	9 (7.08)	2 (1.57)
Psychiatric disorders (*n*=289)	62 (21.45)	41 (14.19)	21 (7.26)

	χ^2^=9.11	χ^2^=4.32	χ^2^=4.43
	p≤0.01	p≤0.05	p≤0.05

During the developmental period, 15.2% of patients with depression, 20% of patients with unspecified psychosis, 15% of those with neurotic disorders and 11.76% of those with other miscellaneous conditions had suffered parental losses. However, these figures were found to be statistically not significant when compared with those of medical patients. Further comparisons in respect of paternal or maternal losses were also found to be not significant in these categories of patients (Table 2). Similarly, in the developmental period, 19.7% cases of schizophrenia, 36.4% cases of bipolar disorder and 33.7% of alcohol dependence syndrome cases had suffered parental losses. However, these figures are found to be statistically significant at various levels when compared with those of medical patients. There is a wide variation in the incidence of paternal losses between various categories of psychiatric patients (range: 5.88%−25.0%). This distribution is found to be non-homogeneous (χ^2^ =13.02; df=6; p<0.05). Maternal deaths (range: 5.88%−8.92%) are evenly distributed (χ^2^ =0.64; df=6; p>0.05). Paternal and maternal losses of 13.6% and 6.0%, respectively, of patients with schizophrenia were found to be not statistically significant; 8.02% of patients with the alcohol dependence syndrome suffered maternal losses (p<0.05). Paternal losses in patients with the alcohol dependence syndrome (25%) and patients with bipolar disorder (27.3%) were found to be highly significant when compared to those of medical patients (Table 2).

**Table 2:** Parental deaths in specific psychiatric disorders compared with general medical conditions

Psychiatric disorder	Loss of father *n* (%)	Loss of mother *n* (%)	Loss of both the parents *n* (%)
Depression (*n*=46)	4 (8.6) χ^2^=0.1	3 (6.5) χ^2^=1.44	7 (15.2) χ^2^=1.63
Unspecified psychosis (*n*=25)	3 (12.0) χ^2^=0.18	2 (8.0) χ^2^=1.32	5 (20.0) χ^2^=2.85
Neuroses (*n*=40)	3 (7.57) χ^2^=0.1	3 (7.57) χ^2^=0.1	6 (15.0) χ^2^=1.34
Miscellaneous (*n*=34)	2 (5.88) χ^2^=0.11	2 (5.88) χ^2^=0.67	4 (11.76) χ^2^=0.04
Schizophrenia (*n*=66)	9 (13.6) χ^2^=2.2	5 (6.0) χ^2^=2.9	13 (19.7) χ^2^=4.85*
Bipolar disorder (*n*=22)	6 (27.3) χ^2^=8.14**	2 (9.1) χ^2^=0.1	8 (36.4) χ^2^=12.9***
Alcohol dependence syndrome (*n*=56)	14 (25.0) χ^2^=11.35***	5 (8.92) χ^2^=3.88*	19 (33.9) χ^2^=18.1***

* p<0.05;

** p<0.01;

*** p<0.001

A comparison of parental loss between a particular category and other psychiatric categories showed that patients with the alcohol dependence syndrome suffered significantly high paternal loss (χ^2^ =6.67; p<0.01). Patients with bipolar disorder suffered paternal loss at a lower level of significance (χ^2^ =3.35; p<0.1). Maternal loss was not a significant factor in any category of patients (Table 3).

**Table 3:** Differences in the incidence of death between a specific psychiatric category and other categories

Psychiatric disorder	Loss of father χ^2^	Loss of mother χ^2^	Loss of both the parents χ^2^
Schizophrenia	0.02	0.72	1.48
Bipolar disorder	3.35*	0.18	3.52*
Depression	1.36	0.73	1.80
Unspecified psychosis	0.10	0.01	0.67
Neuroses	0.1	0.1	1.15
Alcohol dependence syndrome	6.67***	1.8	6.42**
Miscellaneous	0.11	0.67	0.04

* p<0.10;

** p<0.02;

*** p<0.01

## DISCUSSION

Whether bereavement in early life is an important aetiological factor in the development of adult psychiatric disorder is an issue on which no consensus has emerged as yet. This may be largely due to the fact that development is the result of a dynamic process occurring between the invariable genetic factors in an individual on the one hand, and the variable cultural, economic, social and environmental factors, on the other. The deleterious effect of death of the parents can be expected to be offset by matching surrogate care, especially in a society where close-knit family ties are still held with pride. The present study strongly indicates that parental loss is a significant factor in the future development of psychiatric disorders. This is at variance with the findings of Das.^11^ Medical patients in his sample had also shown a high percentage of parental loss. It is possible that this could have been due to poor medical facilities available to parents of soldiers more than a generation ago in India, particularly in rural areas from which most of the soldiers are usually recruited. This confounding factor is perhaps not operational now, as medical facilities, though not yet ideal, have improved over time. While parental losses in patients with medical illness dropped substantially from 27.6% in 1972 to the present 8.7%, a corresponding fall has not occurred in case of psychiatric disorders (34% and 24.5%). Many factors such as common genetic predisposition, late marriage and suicide,^6^ and alcohol-related physical disorders might be responsible for the larger number of parental deaths in those with psychiatric disorders and perhaps have remained substantially unaltered over the years, while medical mortality decreased resulting in increased longevity. Viewed from this angle, even Das's figures now assume a new significance.

The findings of this study are also at variance with those of Tennent *et al*.^8^ Pitts *et al*.,^7^ Granville-Grossman,^6^ and Furukawa *et al*.^9^ Furukawa *et al*.^9^ had exclusively studied cases of the alcohol dependence syndrome in Japan. The workers themselves had commented on the differences in alcohol metabolism between Caucasoid and Mongoloid races. The resistance of Mongoloid races to alcohol dependence is well known. They also allude to the genetic heterogeneity of alcoholism subtypes and the possibility of parental loss being a risk factor for one type and not the other. Though Mongoloid race soldiers are present in the armed forces of India, there were none in the present sample. This differential racial distribution and the possible heterogeneity of subtypes between the Indian and Japanese populations might be responsible for the divergent findings. Tennent *et al*.^8^ had excluded cases of alcohol dependence and schizophrenia from their study. However, they did find a significant difference in the parental losses between community ‘non-cases’ (6.5%) and psychiatric outpatients (15.5%). Outpatients of their sample experienced more paternal deaths. In a subsequent study, Tennent and Bernardi^12^ reported excess parental loss due to separation in cases of alcoholism. Pitts *et al*.^7^ had used diagnostic criteria of various workers including those of Jellinek for alcoholism. They had clubbed all mood manifestations as affective disorder and cases of alcoholism formed only 8.3% of their sample of 748 patients.^7^ In the present study, 19.4% of the cases had alcoholism. It can be reasonably assumed that not all patients of the sample of Pitts *et al*.^7^ were indeed cases of the alcohol dependence syndrome as it is understood now. This particular category alone contributed to 31% of those with total parental losses in the present study. No further comparison could be made with the study of Pitts *et al*.^7^ as they had not given the actual number of parental losses that their patients of alcoholism had suffered. Lastly, Granville-Grossman's sample^6^ consisted entirely of patients with schizophrenia. In their study, non-schizophrenia siblings were used as controls, the rationale of which remains unclear to the present workers. In any case, patients with schizophrenia did not contribute to the parental death load as much as those with bipolar disorder and the alcohol dependence syndrome in the present study. Thus, despite the differences in methodological and diagnostic criteria, the areas of disagreement with the present study and others with negative findings are not absolute.

The sample size of the present study, though small, is comparable to that of some previous studies.^2^,^9^,^11^,^12^ The findings of the present study are broadly similar to those of Dennehy^4^ in respect of schizophrenia, bipolar disorder and the alcohol dependence syndrome, the evidence in relation to the last category being particularly robust. Hilgard *et al*.^5^ and Tennent and Bernardi^12^ also found significant parental losses in patients with alcoholism. Like Dennehy,^4^ the present workers also observed a greater number of parental and maternal losses in alcohol-dependent persons, the significance of the paternal losses being much higher. When comparisons are made between the various psychiatric categories, it is again the loss of father which appears important in case of bipolar disorder (weakly significant) and alcohol dependence (highly significant). It is possible that the security and structure of discipline maintained by the father in traditional Indian households militate against the development of alcohol dependence in the progeny. However, it may not be prudent to infer that paternal death acts exclusively as an environmental deprivation factor as 30% of patients with alcoholism are known to have a history of alcoholism in the family.^13^ Significantly, 33% of the patients with alcoholism in the present study had suffered parental deaths. If these deaths were due to serious alcohol-related physical disabilities or suicides, the present study may in fact point to a genetic factor as a variable of aetiological significance in the alcohol dependence syndrome. However, the workers of the present study have not attempted to ascertain the causes of parental death. None of the earlier workers too had addressed themselves to this task earnestly. The miniscule representation of patients with alcoholism in Das's study (0.5%) precludes the possibility of taking the hypothesis a little further.^11^ Tennent and Bernardi^12^ found that parental losses due to separation were of more significance than those due to death. So this study cannot set at rest the nature–nurture controversy fully.

Parental death does not appear to be an important factor in the development of neurotic disorders. This is at variance with the findings of Raskin *et al*.^14^ and Kendler *et al*.^3^ The findings in case of depression are more significantly at variance with the generally accepted belief, based on the study of Brown *et al*.^2^ that maternal death before the age of 11 years is an important factor for the future development of depression.

It appears from the above that generally the father plays an important mental heath role in the formative years of a man's life, particularly in the Indian setting in case of alcohol dependence. Nothing can be said about women in this regard, as there were no female subjects in this study. Whether the father passes on his healthy (or unhealthy) genes or offers a healthy model to his male offspring is a question that cannot be answered by this simple study. The workers are inclined to agree with Furukawa *et al.*^9^ that cultural factors may be important influences in modulating the effect of parental death but as this study made no attempt to ascertain the nature of the support systems that were available to the hapless patients after the death of either parent, a definitive statement cannot be made in this regard. Comparison with a community sample would also have made the findings more meaningful. The workers propose to undertake a comprehensive investigation of this topic, building upon certain insights gained through this study.
